# Supplementary material for: Human VDAC pseudogenes: an emerging role for VDAC1P8 pseudogene in acute myeloid leukemia
Source: Biol Res. 2023 Jun 22;56:33. doi: 10.1186/s40659-023-00446-1 (PMC10286422; doi:10.1186/s40659-023-00446-1)

Suppl. Fig. 1a-b

a

***VDAC1P8* transcripts in Ensembl database (release 105)**				
**Transcript ID**	**Name**	**bp**	**Biotype**	**Exon n°**
ENST00000610068.5	*VDAC1P8-210*	1251	Processed transcript	5
ENST00000589489.1	*VDAC1P8-204*	997	Retained intron	2
ENST00000438118.6	*VDAC1P8-203*	987	Retained intron	3
ENST00000619849.4	*VDAC1P8-213*	982	Retained intron	4
ENST00000622321.1	*VDAC1P8-214*	902	Retained intron	3
ENST00000612298.1	*VDAC1P8-212*	886	Retained intron	2
ENST00000406025.2	*VDAC1P8-201*	853	Transcribed processed pseudogene	1
ENST00000593045.5	*VDAC1P8-208*	780	Retained intron	3
ENST00000589563.5	*VDAC1P8-205*	752	Processed transcript	4
ENST00000590703.5	*VDAC1P8-206*	752	Retained intron	2
ENST00000415586.5	*VDAC1P8-202*	702	Processed transcript	3
ENST00000593175.1	*VDAC1P8-209*	698	Retained intron	3
ENST00000591189.5	*VDAC1P8-207*	640	Processed transcript	5
ENST00000611810.1	*VDAC1P8-211*	422	Processed transcript	3

b

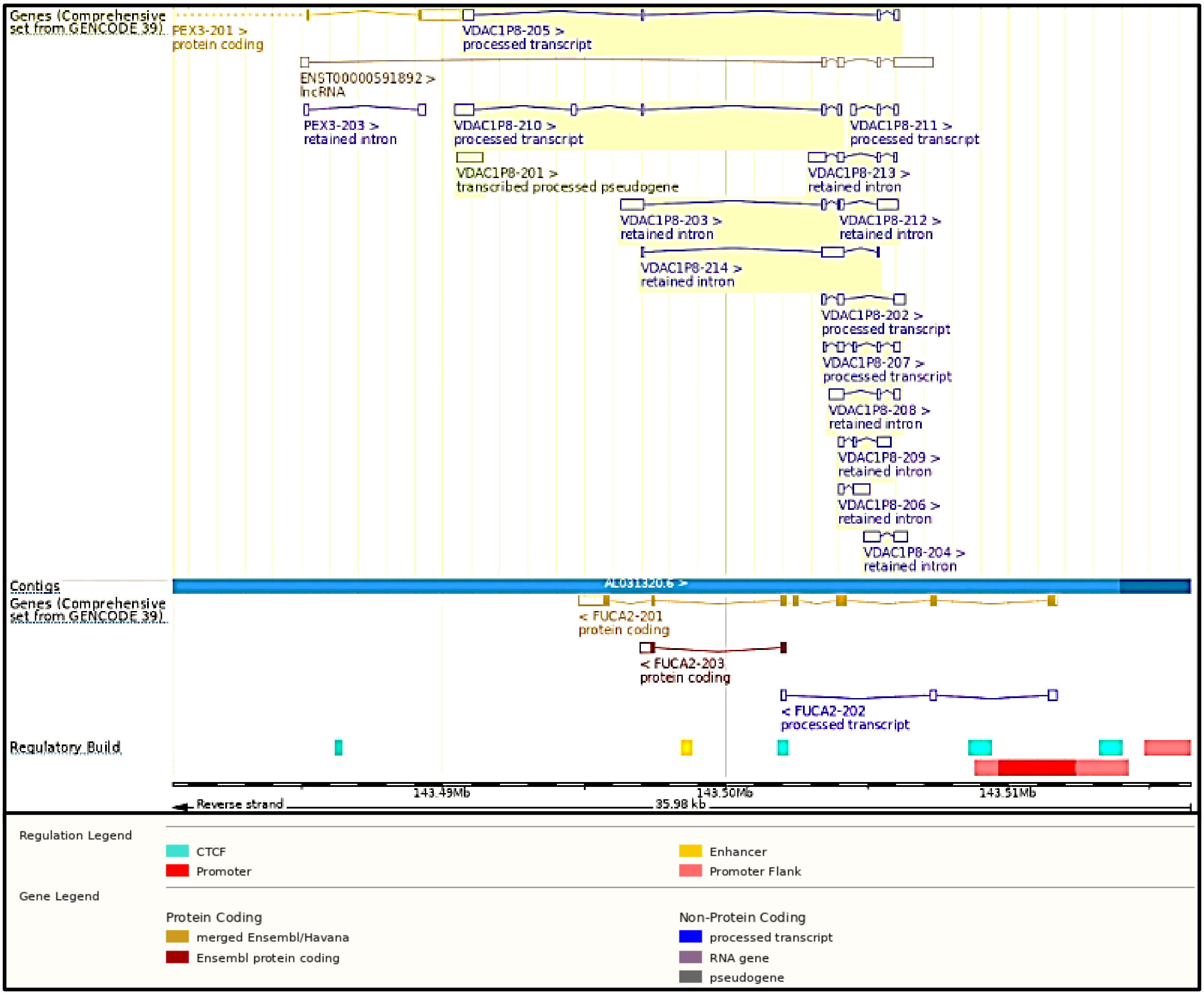

Supplement: Supplementary file 1 — Additional file 1: Figure. S1a, b Summary of annotated transcripts for the VDAC1P8 pseudogene from GENCODE v.39 Ensembl 105. In (a) the table reports gene annotations of 14 transcripts for VDAC1P8 reported on Ensembl database release 105. In (b) the screenshot modified by GENCODE v.39 illustrates some main structural and regulatory features of the variants' alignment. [file 40659_2023_446_MOESM1_ESM.pdf]
