# Supplementary figures and images for: Human VDAC pseudogenes: an emerging role for VDAC1P8 pseudogene in acute myeloid leukemia
Source: Biol Res. 2023 Jun 22;56:33. doi: 10.1186/s40659-023-00446-1 (PMC10286422; doi:10.1186/s40659-023-00446-1)

Suppl. Fig. 2

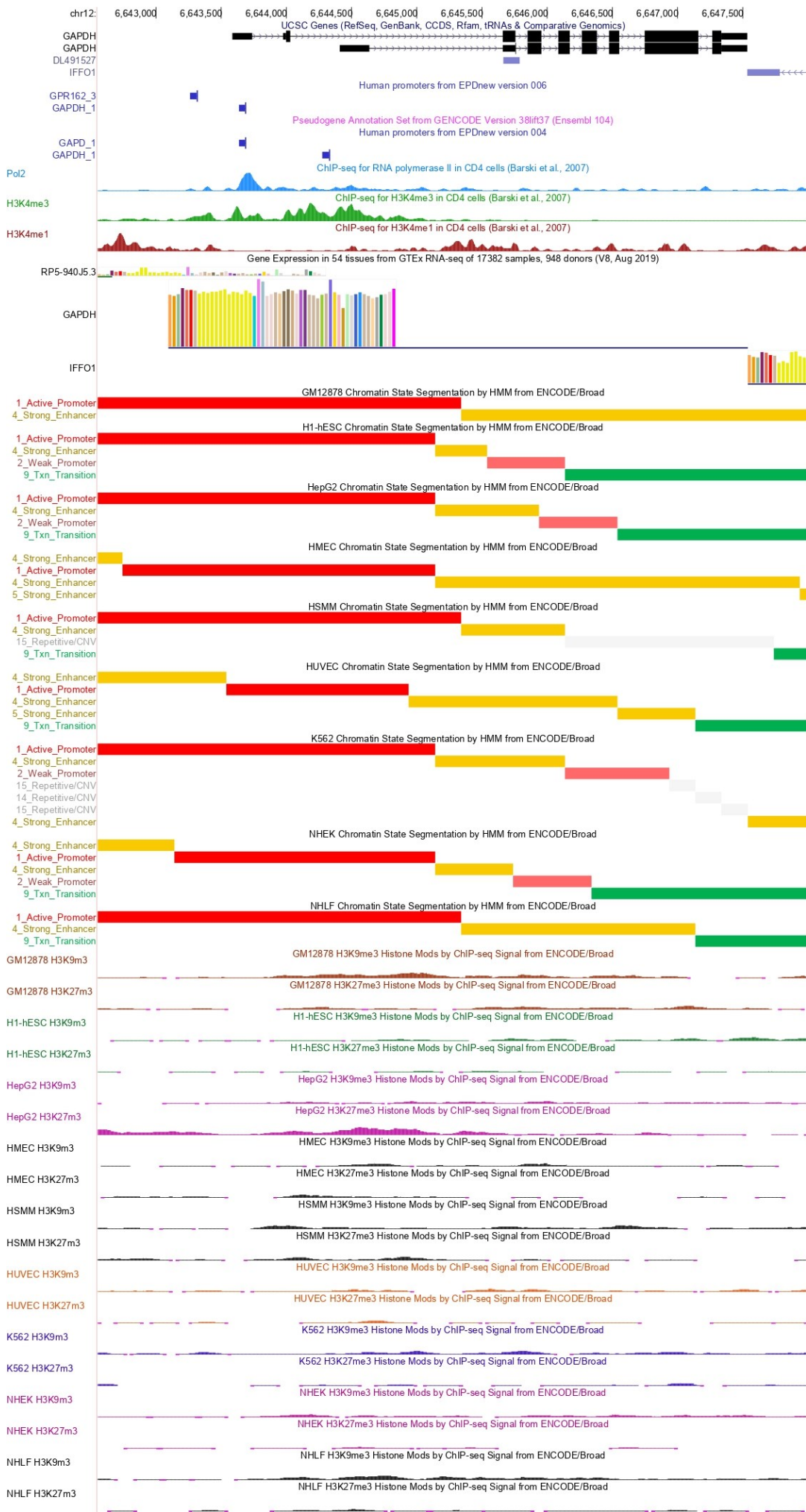

Supplement: Supplementary file 2 — Additional file 2: Figure. S2. Chromatin state and genomic features of GAPDH gene from UCSC Genome Browser GRCh37/hg19. The genomic context of GAPDH set around 1000 Kb upstream and downstream of the annotated Refseq is shown. The selected regulatory hub tracks are Pseudogene Annotation Set from GENCODE v.38lift37 Ensemble 104, Eukaryotic Promoter Database EPD v.4-6, CpG island track, Genotype-Tissue Expression GTEx RNA-seq v.8 2019, ChIP-Seq data for RNA polymerase II, H3K4me3 and H3K4me1, used as markers of transcriptional activation, while H3K9me3 and H3K27me3 are markers of transcriptional repression, and chromatin state segmentation by Hidden Markov Model from the ENCODE/Broad project of nine different cell lines (GM12878, H1-hESC, HepG2, HMEC, HUVEC, K562, NHEK, NHLF) identified using the following different colors: bright red=active promoter; light red= weak promoter; orange = strong enhancer; dark green = transcriptional transition/elongation (Txn). [file 40659_2023_446_MOESM2_ESM.pdf]

Suppl. Fig. 3

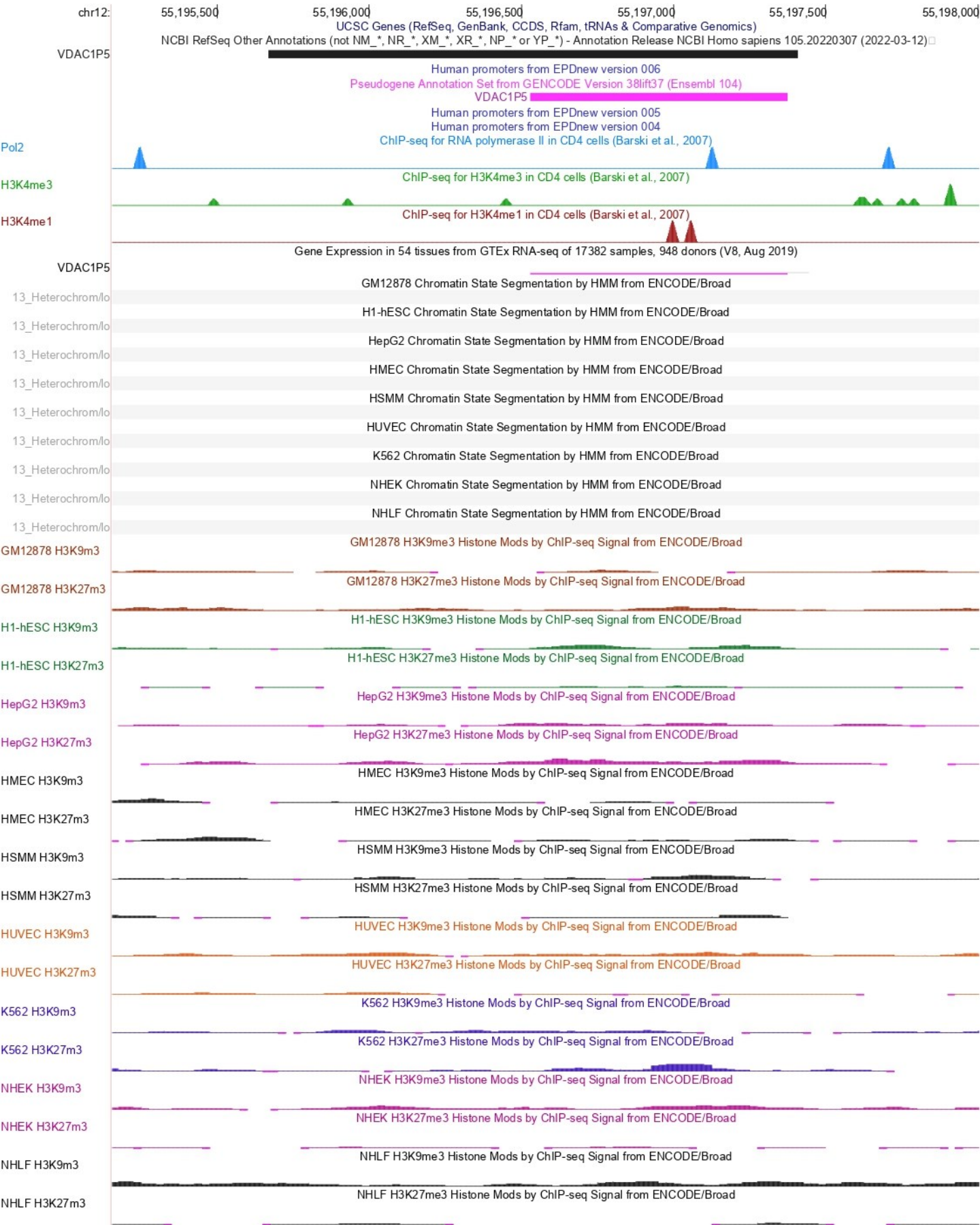

Supplement: Supplementary file 3 — Additional file 3: Figure. S3. Chromatin state and genomic features of VDAC1P5 gene from UCSC Genome Browser GRCh37/hg19. The genomic context of VDAC1P5 set around 1000 Kb upstream and downstream of the annotated Refseq is shown. The selected regulatory hub tracks are Pseudogene Annotation Set from GENCODE v.38lift37 Ensemble 104, Eukaryotic Promoter Database EPD v.4-6, CpG island track, Genotype-Tissue Expression GTEx RNA-seq v.8 2019, ChIP-Seq data for RNA polymerase II, H3K4me3 and H3K4me1, used as markers of transcriptional activation, H3K9me3 and H3K27me3 are markers of transcriptional repression, and chromatin state segmentation by Hidden Markov Model from the ENCODE/Broad project of nine different cell lines (GM12878, H1-hESC, HepG2, HMEC, HUVEC, K562, NHEK, NHLF) colored in grey to indicate the heterochromatin state. [file 40659_2023_446_MOESM3_ESM.pdf]

a

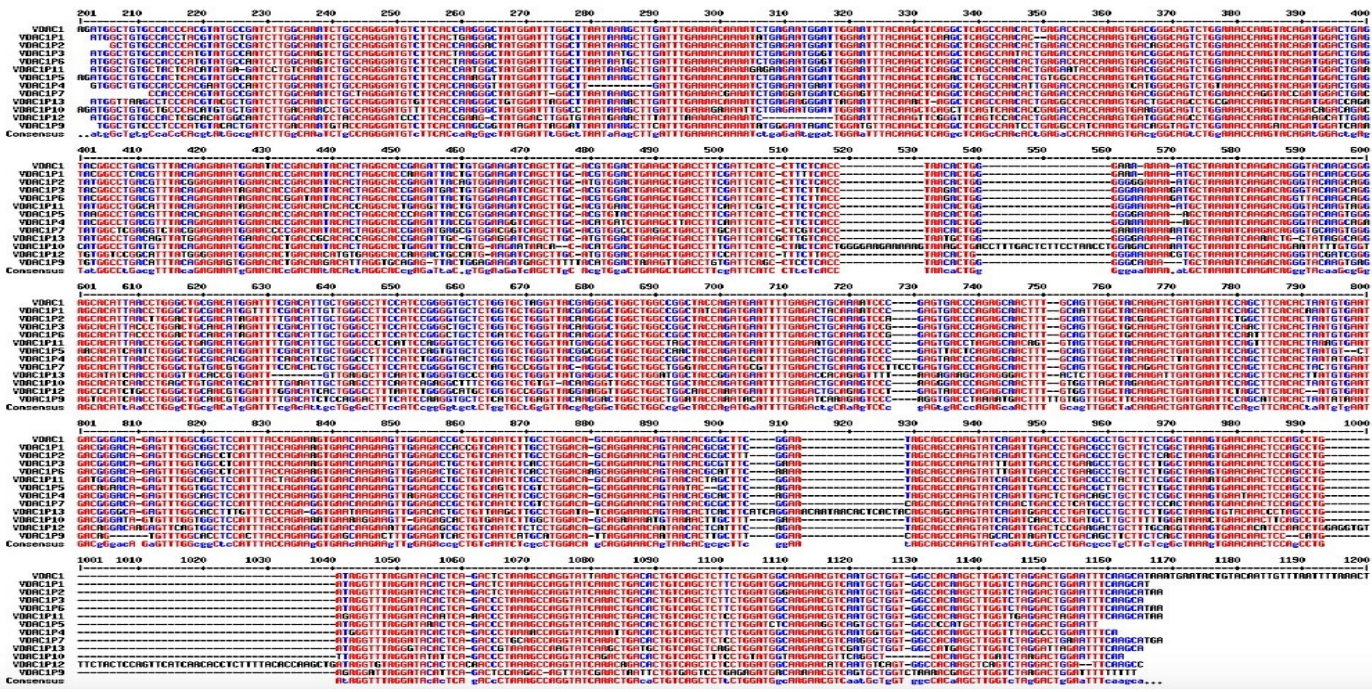

b

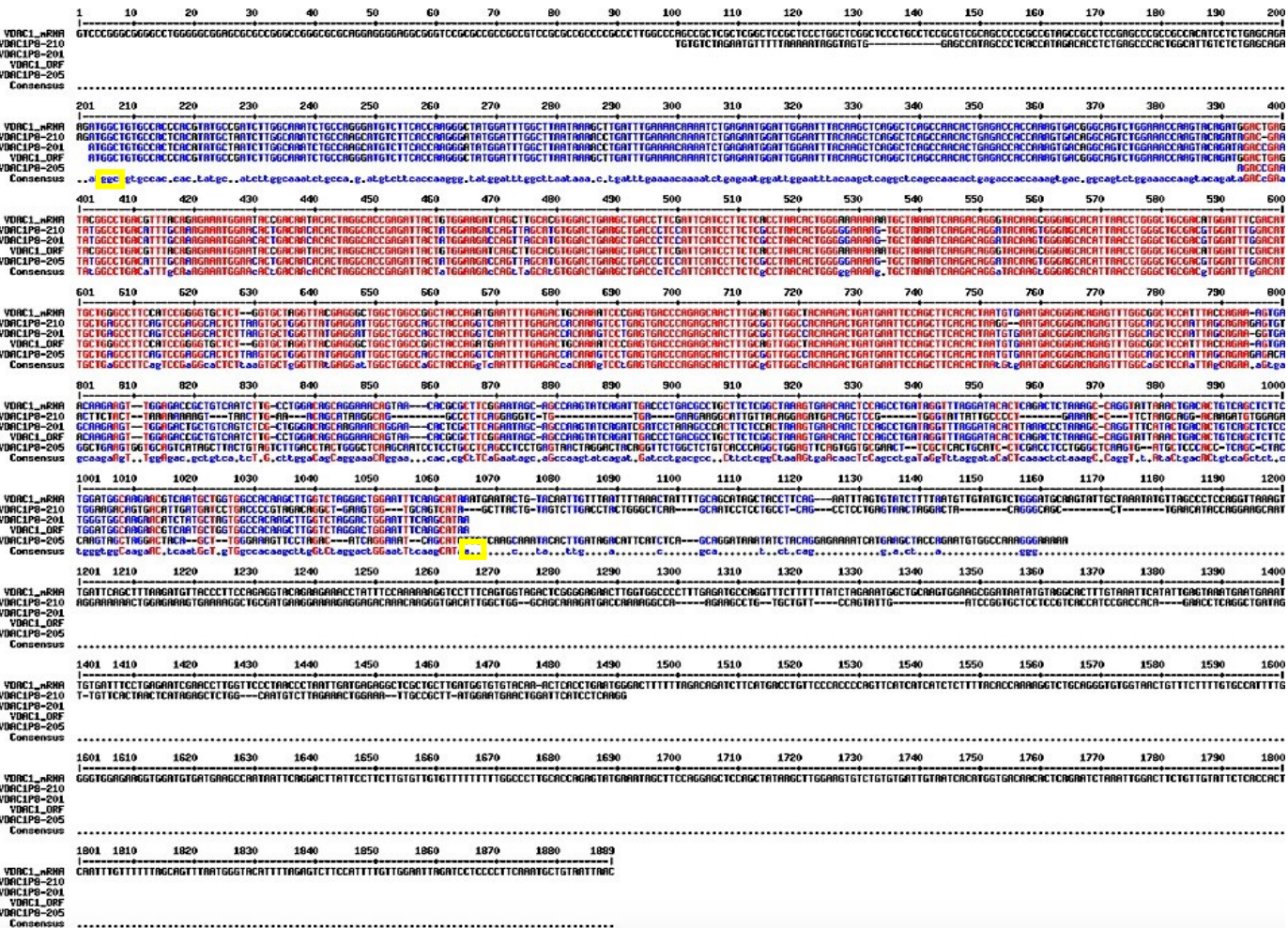

Supplement: Supplementary file 4 — Additional file 4: Figure. S4a, b Sequence multi-alignments among VDAC1 and pseudogenes. In (a) the multi-alignment encompasses VDAC1 against VDAC1P1-7 and VDAC1P9-13. In this picture, the VDAC1 mRNA sequence is shown starting at nucleotide 201 because its start ATG is located at 203-205 position and also because the previous sequence stretches nucleotides 1-200 does not align with the VDAC1 pseudogenes sequences. In (b), the multi-alignment is among VDAC1 and splicing variants of VDAC1P8 VDAC1P8-201, -205 and -210. The start and end codons of the VDAC1 coding sequence are boxed in yellow. [file 40659_2023_446_MOESM4_ESM.pdf]

## Suppl. Fig. 7

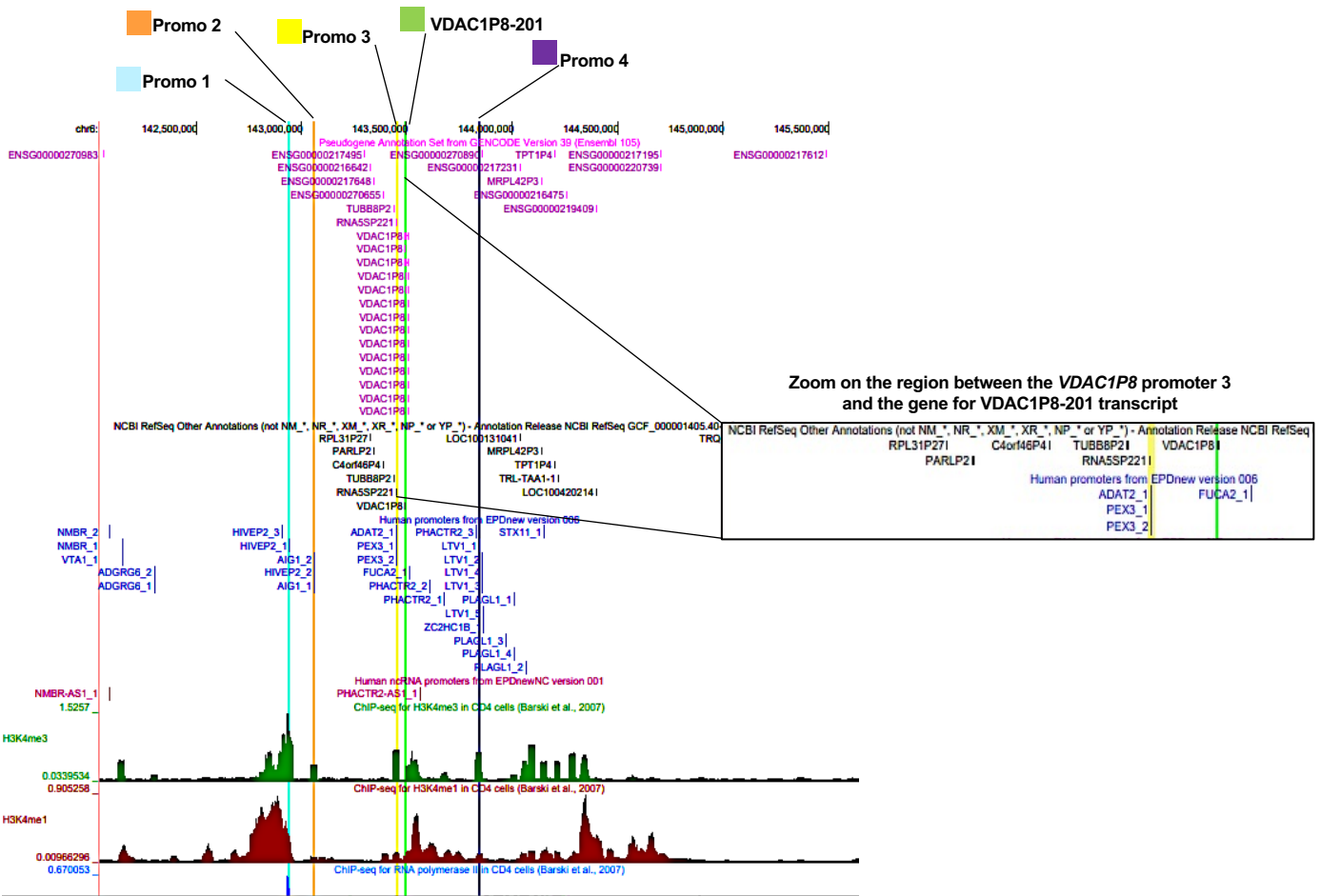

Supplement: Supplementary file 7 — Additional file 7: Figure. S7. Mapping of the best putative promoters for the VDAC1P8 pseudogene (Promo 1-4) inferred by GeneHancer and found in Genecards (hg38). Genomic regions indicated in figure as Promo 1, 2, 3 and 4 correspond to putative VDAC1P8 promoters (GH06J142940, GH06J143449, GH06J143058, GH06J143842) selected from GeneCards (https://www.genecards.org/cgi-bin/carddisp.pl?gene=VDAC1P8) with the best GH score (from 2.1 to 1.9). They map around the NCBI Refseq of VDAC1P8 gene and the transcript VDAC1P8-201 (ENST00000406025.2). All four putative promoters for VDAC1P8 fall within transcriptionally active chromatin regions as indicated by the levels of activating methylation at histone H3 (H3K4m1 and H3K4m3, histone H3 methyl-lysine 4 and histone H3 trimethyl-lysine 4, respectively). Bottom right, an enlargement of the genomic region between VDAC1P8 promoter 3 and the gene for the VDAC1P8-201 transcript, a region potentially under the control of the regulatory sequences of the adjacent genes ADAT2, PEX1 and FUCA2. [file 40659_2023_446_MOESM7_ESM.pdf]
