# Supplementary material for: Human VDAC pseudogenes: an emerging role for VDAC1P8 pseudogene in acute myeloid leukemia
Source: Biol Res. 2023 Jun 22;56:33. doi: 10.1186/s40659-023-00446-1 (PMC10286422; doi:10.1186/s40659-023-00446-1)
Supplement: Supplementary file 6 — Additional file 6: Figure. S6a, b Methylation levels of VDAC1P1, VDAC1P4, VDAC1P8 and VDAC1P11 gene in putative promoter (a) sequence and gene body (b). *The average methylation of different healthy human samples from single-based resolution methylomes (SRMs) are provided by MethBank v.4.1 (https://ngdc.cncb.ac.cn/methbank). SRMs data are calculated as β-Value that reflects the methylation intensity at each CpG site. β-Values of 0–1 (represented from 0 to 1) indicate signifying percent methylation, from 0 to 100%, respectively, for each CpG site. [file 40659_2023_446_MOESM6_ESM.pdf]

Suppl. Fig. 6a-b

a. **Average methylation of *VDAC1, VDAC1P2, VDAC1P4, VDAC1P8 and VDAC1P11* promoter**

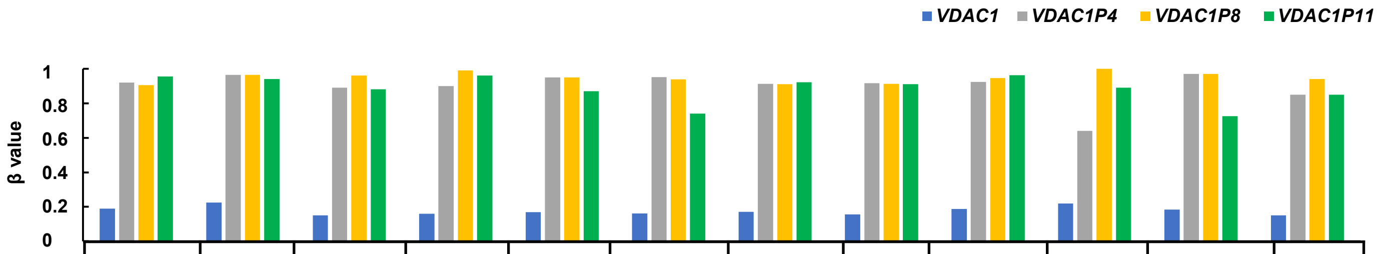

	Adipose	Adrenal gland	Bladder	Breast	Central nervous system	Circulatory system	Digestive system	Lung	Lymphatic system	Placenta	Prostate	Psoas
VDAC1	0.19	0.23	0.15	0.16	0.17	0.16	0.17	0.16	0.19	0.22	0.19	0.15
VDAC1P4	0.92	0.97	0.89	0.9	0.95	0.95	0.91	0.92	0.92	0.64	0.97	0.85
VDAC1P8	0.91	0.97	0.96	0.99	0.95	0.94	0.91	0.91	0.95	1	0.97	0.94
VDAC1P11	0.96	0.94	0.88	0.96	0.87	0.74	0.92	0.91	0.96	0.89	0.73	0.85

b. **Average methylation of *VDAC1, VDAC1P2, VDAC1P4, VDAC1P8 and VDAC1P11* gene body**

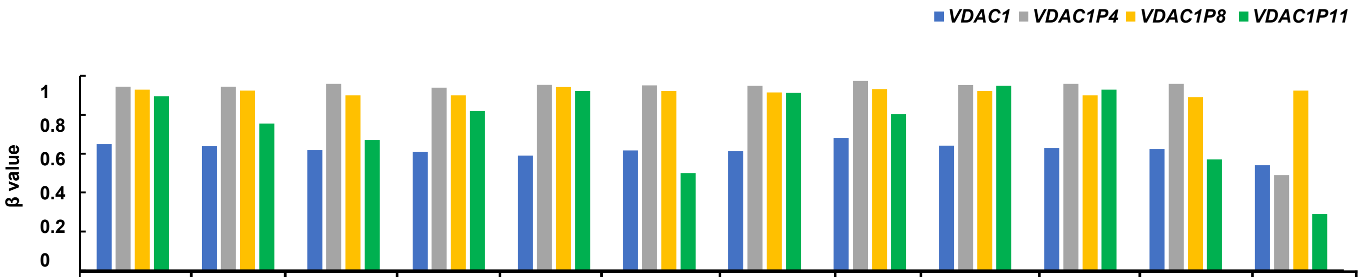

	Adipose	Adrenal gland	Bladder	Breast	Central nervous system	Circulatory system	Digestive system	Lung	Lymphatic system	Placenta	Prostate	Psoas
VDAC1	0.65	0.64	0.62	0.61	0.59	0.62	0.61	0.68	0.64	0.63	0.63	0.54
VDAC1P4	0.95	0.95	0.96	0.94	0.95	0.95	0.95	0.97	0.95	0.96	0.96	0.49
VDAC1P8	0.93	0.93	0.9	0.9	0.94	0.92	0.92	0.93	0.92	0.9	0.89	0.93
VDAC1P11	0.9	0.76	0.67	0.82	0.92	0.5	0.91	0.8	0.95	0.93	0.57	0.29
