# Supplementary material for: Human VDAC pseudogenes: an emerging role for VDAC1P8 pseudogene in acute myeloid leukemia
Source: Biol Res. 2023 Jun 22;56:33. doi: 10.1186/s40659-023-00446-1 (PMC10286422; doi:10.1186/s40659-023-00446-1)

Suppl. Fig. 5

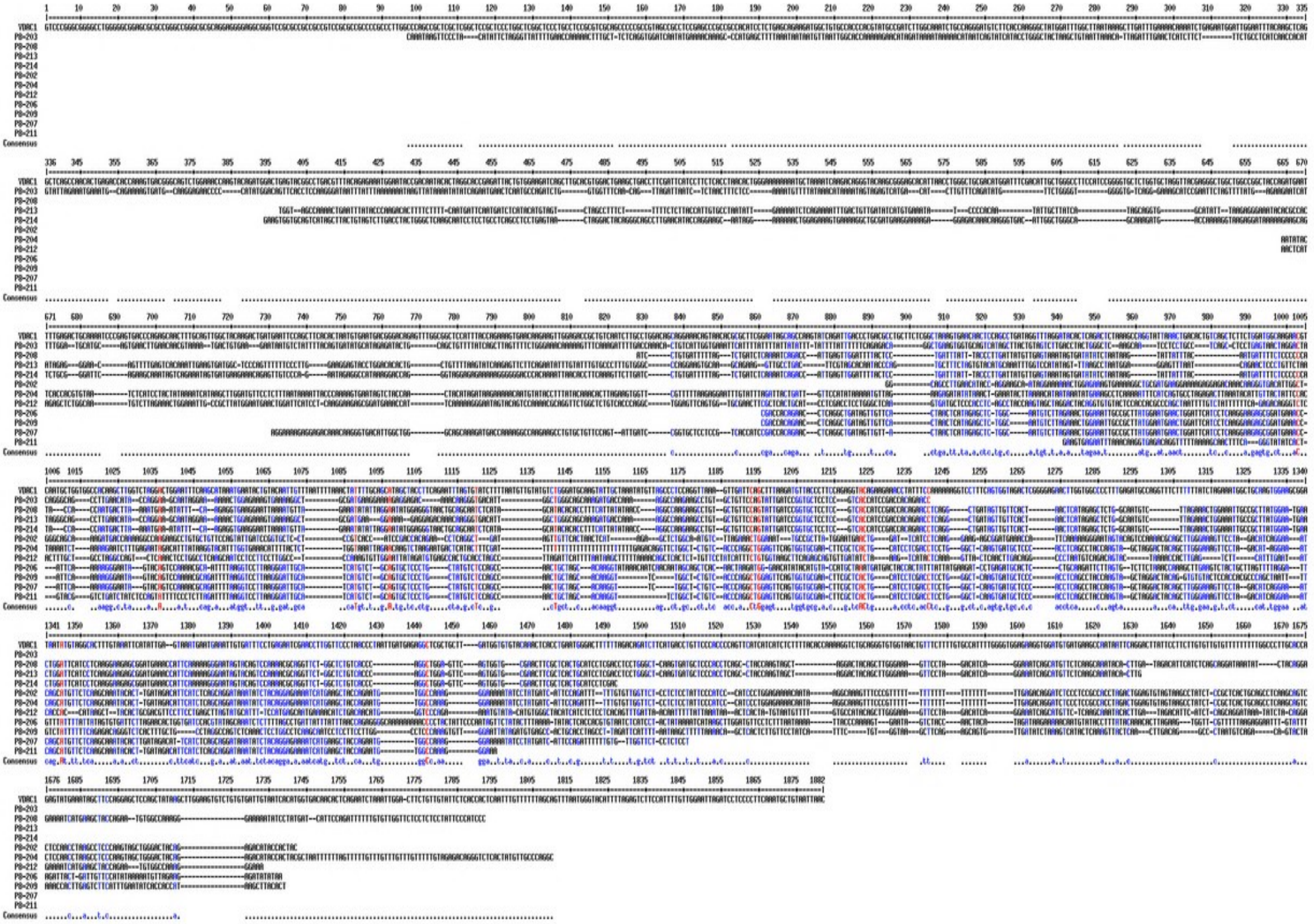

Supplement: Supplementary file 5 — Additional file 5: Figure. S5. Multi-alignment between the VDAC1 mRNA and some VDAC1P8 alternative transcripts sequences. Alignment achieved by http://multalin.toulouse.inra.fr/multalin/ setting the default parameters. The regions with high consensus value (90% setting) are shown in red, while regions with low consensus value (50% setting) are shown in blue. [file 40659_2023_446_MOESM5_ESM.pdf]
